# Social accountability: Attitudes and awareness among undergraduate medical students in Morocco 

**DOI:** 10.30476/jamp.2020.87197.1298

**Published:** 2021-01

**Authors:** MAJDA SEBBANI, LATIFA ADARMOUCH, ADIL MANSOURI, MOHAMED AMINE

**Affiliations:** 1 Community Medicine and Public Health Department, Research laboratory, Biosciences and Health, School of Medicine, Cadi Ayyad University, Marrakech, Morocco; 2 Clinical research unit, Mohammed VI university Hospital, Marrakech, Morocco

**Keywords:** Social accountability, Medical students, Perceptions

## Abstract

**Introduction:**

As future health professionals, medical students should be aware of their social accountability and their role in the society. The aim was to assess the attitudes and awareness of medical students regarding the social accountability and to identify the factors determining their attitudes and possible levers for action.

**Methods:**

Online survey among undergraduate students at the medical school in 2019 (N = 2128). The data collection tool was developed based on the literature review, the principles of social accountability SR and the toolkit (The development of the Students' Toolkit on Social Accountability of Medical Schools was a collaboration between the International Federation of Medical Students' Associations (IFMSA) and the Training for Health Equity Network (THEnet). The data were collected in December by LimeSurvey software version 1.90 and analyzed by SPSS version 16 according to the usual descriptive and bivariate. The Fisher test was used to compare two percentages and the Student t-test to compare two means on two independent samples (significance level was 0.05). The verbatims were grouped and analyzed.

**Results:**

271 students participated (response rate = 12.7%). The mean age was 20.6±2.6 years (N = 257). The Female/Male ratio was 1.85. Only 33.5% had heard of social accountability. It was linked to “commitment to the community” in 75.1% of cases, “to be a good citizen” in 66.1% and “to be responsible for one's actions” in 56.4%. Nearly 79% thought that students did not really have a role in society and that they should focus on their studies. Being a member of an association was a factor determining the positive attitude towards the SA (82.1% versus 65.2%, p=0.031). Students believed that the school had some strategies of social accountability. The results of the verbatim emphasize the need to multiply the practices and opportunities for interaction with the social environment for students.

**Conclusion:**

The level of knowledge of the concepts by the students seems to be average. It would be necessary to look for the means to plead in favor of reinforcing our school strategies to concretize the principles of SR while training the students.

## Introduction

In medical education, social accountability (SA) of academic institutions has been defined by the World Health Organization as "the obligation to direct their education, research and service activities towards addressing the priority health concerns of the community, region and/or nation that they have a mandate to serve"( 1 ). The faculty of medicine is compared to a “company” that produces human resources in health. Therefore, it is its social accountability to instill the principles of SR in future doctors during and throughout the training. According to the available literature, the concept of SA seems to be poorly understood by both trainers and students. The students - real human engines for achieving health goals in the coming decades - should be an agent of change. In 2010, the Global Consensus proposed ten areas of change for health training institutions to become socially responsible ( 2 ). The concept encourages schools to take an external look at the changing needs of society and to examine how medical practice can have a positive impact on the health of the population; family medicine thus comes to embrace the concept of social accountability ( 3 ). There are also several models for integrating social responsibility into training curricula, but it should be established more as a culture among students and professors ( 4 ).

Since 2012, an international research-action project on social accountability of French-speaking medical faculties using a pragmatic approach has emerged ( 5 ). Several projects at the FMPM to concretize actions demonstrated the commitment among the 44 faculties of medicine partners of this international project. These actions were crowned by the accreditation in 2019 by the CIDMEF (International Conference of Deans and Faculties of Medicine of French Expression). The literature is rich in publications that deal with the values ​​of SR and the link of SR with medical education; however, research on SR is scarce. Among the main results of the first phase of the international French-speaking project, which was concerned with assessing the perception and knowledge of SR, the ignorance of the concepts and principles of SR and the important role of students as leverage and opportunity for better consideration of the faculties of the principles of SR appeared ( 5 ). Indeed, students represented 20.8% of the 1291 participants in the survey among different profiles at the schools involved in the project. The social responsibility of the faculties of medicine is considered as a means to reconcile the students with their medical commitment ( 6 ). The degree of integration of this concept by the faculties of medicine is a criterion of excellence although its measurement remains difficult. In this sense, the WHO has established a grid based on the evaluation of four values ​​in the field of research training and services (quality, relevance, efficiency, equity) ( 1 ). The translation with the students will be done during the training and after it during the medical practice. During the training, it is important to know what the students think about it, what their knowledge and representations on social responsibility are and how they view the engagement of their faculty in the realization of this concept. This sort of internal assessment would continuously provide action levers for better consideration of SR within the medical school. The aim was to explore the students’ perceptions, awareness, and understanding of the concepts and values expressed within social accountability and identify factors and areas for improvement.

## Methods

This cross-sectional observational study used a self-administered electronic questionnaire. The data collection tool was developed based on the literature review, the principles of social accountability and the toolkit [The development of the Students' Toolkit on Social Accountability of Medical Schools was a collaboration between the International Federation of Medical Students' Associations (IFMSA) and the Training for Health Equity Network (THEnet)] ( 7 ). It included 3 types of questions:

▪ Questions with binary answers (yes or no) or multiple choices.
▪ A score calculated from the responses according to a Likert scale of 4 (0 = no, 1 = a little, 2 = average, 3 = Excellent) and a 4th response modality = I don't know. The overall assessment score is interpreted according to the following intervals:
  o 0 to 8: Start a conversation with your classmates and school to build social accountability at your school.
  o 9-17: Your school has some social accountability strategies, look for ways to advocate building on these existing strategies.
  o 18-26: Your school is doing well; look for areas of weakness and ways to advocate improving social accountability.
  o 27-36: Your school has a strong foundation in social accountability; advocate for continued growth and leadership in social accountability
▪ Open-ended questions for collecting verbatims and suggestions from participants.

The electronic form was structured in 3 sections: 1) socio-demographic characteristics of the participants, 2) knowledge of the concept of SAR, 3) perceptions and attitudes of the social role of students, 4) Perceptions and evaluation of the social responsibility of the medical school by its students.

The 15 min questionnaire was tested with 3 students before it was distributed. Students were contacted through the institutional electronic platform and were invited to participate via a link to the school assessment database (LimeSurvey version 1.90). Three reminders were sent by email through the representatives of the students and via social media. Data collection took place between December 05, 2019, and January 09, 2020.

The quantitative data were extracted by Excel and then analyzed by Statistical Package for the Social Sciences software (SPSS), version 16. The answers to the open-ended questions were grouped and then analyzed according to the content analysis method.

Descriptive results were presented as numbers and percentages and means with standard deviation. The Fisher test was used to compare two percentages and the Student t-test to compare two means on two independent samples to compare age. The statistical significance level was 0.05.

## Results

### Description of the participants

Out of a total of 2,128 students enrolled in the medical school in 2019, 271 participated in the survey. This corresponds to a response rate of 12.7%. Fourteen incomplete observations were excluded during the analyses. The mean age was 20.6±2.6 years old (N = 257). The female/male sex ratio was 1.85 (Table 1).

**Table 1:** Characteristics of the participating students

Variable	Number (n)	Percentage (%)
Study year (N=255)		
First	74	29.0
Second	52	20.4
Third	31	12.2
Fourth	30	11.8
Fifth	27	10.6
Sixth	17	06.7
Seventh or preparing medical thesis	24	09.4
Gender		
Male	90	35.0
Female	167	65.0
Extracurricular activities		
Yes	74	28.8
No	183	71.2
Member of an association		
Yes	56	21.8
No	201	78.2

**Figure 1:**
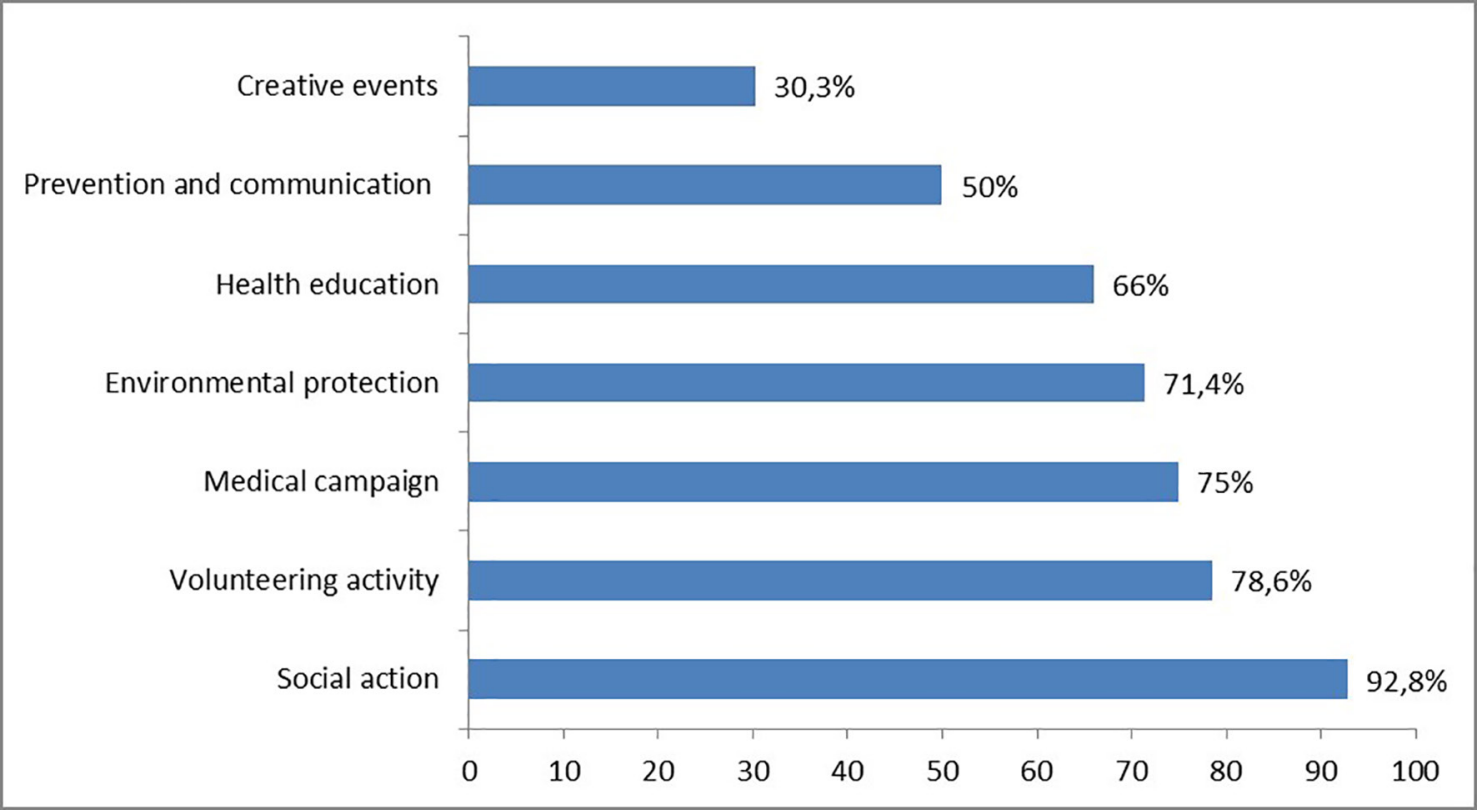
Associative activities for the members of an association during the study period (N=56)

The fields of students’ associations activities were represented by social action in 92.8%, volunteering in 78.6% and participation in medical community outreach medical activity in 75% of the responses (Figure 1).

### Students' knowledge and definitions of the concept of SA

Only 33.5% had heard of social accountability. It was linked to “commitment to the community” in 75.1% of cases. There was no statistical difference between the study cycles (First and second cycle) except for "doctor’s medical responsibility" more reported by pre-clinical (Second cycle) students (p=0.034) (Table 2).

**Table 2:** Students’ knowledge and definitions of Social Accountability (SA)

Students’ Knowledge and definitions of SA	Total n (%)	1^st^ cycle of medical studies (N=126) n (%)	2d cycle of medical studies (N=129) n (%)	P*
Already heard of SA	86 (33.5)	40 (31.7)	46 (35.7)	0.299
Commitment to the community	193 (75.1)	94 (74.6)	98 (76.0)	0.457
Being a good citizen	170 (66.1)	90 (71.4)	79 (61.2)	0.056
Being responsible for one’s action	145 (56.4)	76 (60.3)	68 (52.7)	0.136
Doctor’s medical responsibility	99 (38.5)	56 (44.4)	42 (32.6)	0.034
School’s responsibility regarding the students	88 (34.2)	42 (33.3)	46 (35.7)	0.398

*P value=extremely statistically significant

### Perceptions and attitudes of the social role of students and associated factors

As for the question of social responsibility for students; 26.1% did not know if it was applicable to them and 4.7% answered "no". In fact, 79.3% thought that students did not really have a role in society and that they should focus on their studies.

Having previous knowledge of SR and being a member of an association were the determining factors for the positive attitudes towards the social responsibility of students (p = 0.031 and p=0.035 successively) (Table 3). 

**Table 3:** Factor associated to positive attitudes towards students’ social accountability

Determinants	Student has a social accountability		p*
Yes N=175	n (%)	No or I dont know N=80 n (%)
Gender			
Female	120 (71.9)	47 (28.1)	0.103
Male	57 (63.3)	33 (36.7)	
Study level			
First cycle	81 (64.3)	45 (35.7)	0.09
Second cycle	94 (72.9)	35 (27.1)	
Association’s member			
Yes	46 (82.1)	10 (17.9)	0.01
No	131 (65.2)	70 (34.8)	
Already heard of SA			
Yes	66 (76.7)	20 (23.3)	0.035
No	111 (64.9)	60 (35.1)	
Associative activities			
Medical missions	32 (76.2)	10 (23.8)	0.175
Environmental protection	29 (72.5)	11 (27.5)	0.367
Social action	43 (82.7)	9 (17.3)	0.010
Volunteering	38 (86.4)	6 (13.6)	0.003
Health education	31 (83.8)	6 (16.2)	0.023
Prevention and communication	23 (82.1)	5 (17.9)	0.078
Age (mean±standard deviation in years)	20.65±2.7	20.42±2.3	0.5

*P value=extremely statistically significant

### Perceptions and assessment of the medical school social accountability

The average assessment score for SA in the medical school by the students was 16.14±7.5. Students believed that the faculty had some strategies of social accountability (Table 4).

**Table 4:** Surdents’ perceptions about the medical school social accountability

The statements	Number (n)	Percentage (of agree) (%)
The faculty of medicine has to identify the health needs of the population and to take them into account in the curricula.	230	89.5
Performing an international internship is important for the student to develop an idea about global health.	229	89.1
Medical doctors should participate in community activities.	224	87.2
The faculty of medicine has a formal obligation to teach students the importance of advocacy (defending the rights and interest. social actions).	182	70.8
Medical studies limit students from having para-curricular activities (volunteering. association work. etc.).	160	62.3
Medical students must be concerned with the priority health needs of the local population.	135	52.5
Medical students should focus their learning specifically on local priority health needs.	82	31.9
Priority health needs do not concern physicians; they concern administrative or government professionals.	72	28.0
The faculty of medicine does not have a duty to cultivate the culture of altruism among students.	37	14.4
Being a doctor is a mission towards patients. It does not involve a concern for the community needs.	36	14.0
Students don't really have a role (contribution) in society and they should focus on their studies.	21	08.2

### Verbatim analysis

The results of the verbatim analysis emphasized the different definitions of SA given by the students which can be grouped into categories related to:

The dimension of quality of medical studies: in fact, the students viewed themselves into a quality medical practice, which would serve to solve the problems of society from a posture of student engaged in their current training:

"We must focus in studies so that tomorrow, as doctors, we will be able to diagnose our patients well; otherwise, we will commit crimes against our society", "Studying well to be a good doctor to develop and improve the health system in general", "My responsibility is to work hard so that my society benefits from my knowledge".

The dimension of openness to the environment and the need for more interaction with the community for concrete actions that would reflect the integration of the values ​​of SA during the training: 

"The medical student must feel the needs of the population because he must understand them and our society in particular", "To inform, to educate, to raise awareness ...".

The dimension linked to the quality of the future practitioner and to the values ​​of SR during the practice of the medical profession testifying to the behavior of the future graduate towards his society and the consideration of the global approach to health and the different determinants of health: 

"Respect", "mutual assistance", "citizenship", "humanism", "respect for the Hippocratic oath", "respect for the rules of ethics", "equity", "honesty".

 "Being competent", "Meeting patient expectations", "Responsibility towards patients is included in that towards society", "Caring for people with all possible means without reserve!", "Delivering services and benefits of a good level and adequate to the needs of society", "caring for the physical and mental problems without forgetting the social aspect".

To improve the integration of the SA concept in the school, the students raised the need to multiply the types of practices, the training fields and the opportunities for interaction with the social environment in addition to the space to be given to communication around SR and the diversification of training programs including community outreach medical activity and development activities for students: 

"I favor exchanges with other international universities, and also medical missions", "The role of a doctor is not limited to curing the disease but also is a social role because a doctor is indeed a public health professional."; "I think that we, the students of the medical school, are lucky because we benefit from good quality education, however, we are far from gaining practical experience and interacting with the social environment, which makes our studies very boring."; "I really hope that an extra-curricular activity would be part of the weekly schedule in order to encourage the students to join it, whether it be a sporting or artistic activity or associative".

## Discussion

We highlighted the attitudes and perceptions of the students and their assessment of SR in our faculty. SR assessment is now part of the criteria for medical school accreditation ( 8 ). In this sense, the medical school was accredited in 2020. However, despite the external recognition of excellence, the integration of the students' vision at the center of improving training is essential to be taken into consideration continuously.

One of the main results of this work is the lack of knowledge of what SA represents for a student in training. A small proportion of students have already heard of SA (33.5%). The concept of social responsibility seems to be insufficiently emphasized by training curricula regardless of the level of study of the students. Indeed, one would have imagined that the perceptions would be improved with the approach of graduation. But we did not find any difference between the training cycles except for the "medical responsibility of the doctor," more reported by pre-clinical students. This can be explained by the lack of contact with the community with a narrower medical and clinical vision. In addition, the SA was attached to the commitment to the community and to be a good citizen responsible for his actions. The students also discussed the values ​​of SR such as "quality", "equity", "ethics", "humanism", and "globality".

The authors report similar results across the literature. Although medical students do not fully understand the missions or functions of a socially responsible health professional, they recognize its importance, especially for a recognized definition of the skills expected to meet the needs of society ( 5 ).

The concept does not seem to be familiar to students especially those who do not have social activities alongside studies. The majority of students thought they should focus on their studies, unlike students who have knowledge and practice of a social outreach activity. This would amply justify the need to integrate community outreach activities during the training course. This was suggested by the students as a means of improving the integration of SA into the medical school. This recommendation also emerges when the students' average assessment score for SA in the school was 16.14±7.5. This result would mean that the students thought that the Faculty had some strategies of social accountability and it would, therefore, be necessary to look for means to advocate in favor of strengthening these strategies and building on them.

However, regarding the lack of assessment methods reflecting and measuring the values underpinning social accountability of medical schools ( 9 ), we used the tools available after the literature review that we adapted to our context. The World Health Organization suggests that four principles delineate social accountability--relevance, quality, cost-effectiveness, and equity. Medical schools are evaluated according to their planning, doing, and impact in relation to these principles. THEnet group of medical schools use a shortened version of Boelen and Woollard's framework with 20 criteria to evaluate their programs ( 8 , 10 ).

While studies in other contexts have different qualitative or quantitative methodologies, the results seem to overlap. Social accountability was not a familiar concept for students at Makerere College of Health Sciences in sub-Saharan Africa. However, the respondents contended that it is the individual's responsibility to be 'sensitive' to the needs of the communities the individual serves ( 11 ). Another study conducted among 237 pre-clinical medical students in a medical college in South India found that 61.6% were not aware of their social accountability and 38.4% expressed self-centeredness ( 12 ).

McCrea ML, et al. conducted a qualitative study (focus group) among students nearing graduation (from a five-year undergraduate course) in the UK (2014). The authors concluded that medical students reflect limited awareness of the concept of Social Accountability. While many aspects of the undergraduate training should contribute to the acquisition of those key characteristics of social accountability, these would appear to be underdeveloped and not recognized by students ( 13 ). 

### *Suggestions*

The challenge to medical schools to embed social accountability concepts within graduates requires addressing not only curricular content but also educational approach and medical teaching needs to contain the social world view embedded in the science taught ( 14 ). It is also important to involve information and training not only of students but also of professors within the framework of real training programs ( 15 ). The motivation for social accountability should be reflected in future development programs, program planning and training of general practitioners ( 16 ).

It would be necessary to look for the means to plead in favor of reinforcing our school strategies to concretize the principles of SR while training its students. However, more studies are needed with a longitudinal approach (like cohort) to assess the social accountability practices of the medical students after their graduation.

### *Advantages and limits*

Despite the limited representativeness of the sample of respondents, the results showed that information and awareness are essential and must be reinforced among students. Appropriate approaches to awareness-raising and training have been recommended. Discussions were initiated with students who showed a keen interest in a SA capacity building project during the training, including representatives of students’ associations. The authors have initiated a reflection with the deanship as well as other partners on the possibilities of strengthening the social accountability of students.

This study is integrated into institutional projects to improve the quality of training and the opening of the medical school to its environment. This was a cross-sectional study among a sample of volunteers. There is no evidence to support that the awareness, beliefs and attitudes of students, actually impact how they practice in their future careers. They can change throughout medical education or after graduation. Indeed, even if the medical school intends to train socially responsible graduates, there is no guarantee that they will become socially responsible practitioners ( 17 ).

The low response rate is a limitation in our study due to the electronic method of data collection. This limitation is described in the literature for online surveys. In addition to the small sample of participants reached after several reminders, we thought that social desirability bias should be taken into account when reading the results because of the declarative nature of the responses. This bias could have impacted the responses (overestimation or underestimation).

## Conclusion

The level of knowledge of the concepts by the students seems to be average. The results show the lack of knowledge of what SA represents for a student in training. A small proportion of students had already heard of the SA. The concept seems to be insufficiently emphasized by training curricula regardless of the level of study of the students. Their perceptions raise the importance of more communication and enhance awareness of the concepts and values ​​of social accountability. 
